# Comorbidities in extracorporeal membrane oxygenation: a comorbidome-based analysis

**DOI:** 10.3389/fmed.2026.1829427

**Published:** 2026-06-08

**Authors:** Henry Robayo-Amortegui, Juan Hernández-Puentes, Michel Pérez-Garzón, Eduardo Tuta-Quintero, Estefanía Giraldo-Bejarano, Eliana Lozano-Torres, Carlos Granados-Burgos, Alejandro Quintero-Altare, Tatiana Jaramillo, Alirio Bastidas-Goyes, Gabriel Malagón-Tarqui, Claudia Poveda-Henao

**Affiliations:** 1 Department of Critical Care Medicine, Extracorporeal Life Support Unit, Fundación Clínica Shaio, Bogotá, D.C, Colombia; 2 School of Medicine, Universidad de La Sabana, Chía, Colombia

**Keywords:** comorbidities, comorbidome, critical care, ECMO, risk factors

## Abstract

**Introduction:**

Extracorporeal membrane oxygenation (ECMO) is an advanced life support modality associated with global mortality rates ranging from 48.7 to 76.38%. However, a significant knowledge gap remains regarding the specific impact of comorbidities on patient prognosis, particularly within middle-income settings in Latin America, which are characterized by substantial underreporting.

**Objective:**

To assess the relationship between comorbidities and 30-day mortality in patients receiving ECMO support, distinguishing between venoarterial (VA) and venovenous (VV) configurations.

**Methodology:**

A cross-sectional cohort study was conducted on 436 adult patients requiring ECMO support at a high-complexity institution in Colombia between 2019 and 2024. Comorbidities were assessed using the Charlson Comorbidity Index (CCI). The association with mortality was quantified using Odds Ratios (OR), and prevalence was visualized using a “Comorbidome”.

**Results:**

A total of 420 patients were included, with a mean age of 43.7 (SD:13.0) years and an overall mortality rate of 30%. In the overall ECMO cohort, the most frequent comorbidities were anemia (47.7%), obesity (32.1%), and metabolic disorders (22.0%). Regarding cannulation strategies, VA configuration accounted for 36% of cases, while VV accounted for 64%. Conditions most strongly associated with overall mortality included human immunodeficiency virus (HIV) infection (OR 3.60), chronic kidney disease (OR 2.13), and hypertension (OR 1.68). Mortality associations were modality-specific: in VA-ECMO, chronic kidney disease and cancer presented the highest odds (OR 2.92), whereas in VV-ECMO, HIV infection showed the strongest association (OR 2.44).

**Conclusions:**

Pre-existing comorbidities, particularly HIV, chronic kidney disease, and hypertension, are strongly associated with mortality in this ECMO population. Identifying these conditions and their modality-specific differences is crucial for optimizing candidate selection and guiding clinical management in Latin American intensive care units.

## Introduction

Extracorporeal membrane oxygenation (ECMO) is an advanced life support modality indicated for patients with severe respiratory or cardiac failure refractory to conventional therapies, as well as in specific clinical contexts such as transplantation or durable mechanical support (1). Over the past few decades, its utilization has increased significantly in both adult and pediatric populations, and it is currently available in approximately 6,000 intensive care units in the United States (2). Despite improvements in survival for conditions such as acute respiratory distress syndrome (ARDS) and cardiogenic shock, ECMO-associated mortality remains elevated, ranging from 48.7 to 76.38%, and is influenced by multiple factors, including patient comorbidities (3–5).

Between the two modalities, venovenous (VV) ECMO provides respiratory support, whereas venoarterial (VA) ECMO offers both cardiac and respiratory support. Indications for its use may be influenced by various comorbidities, which constitute a key determinant of prognosis in critically ill patients (6, 7). These include renal or hepatic insufficiency, chronic lung disease, smoking history, advanced age, and history of transplantation, all of which are associated with an increased risk of complications such as hemorrhage, nosocomial infections, peripheral ischemia, and neurological events (8, 9). These pre-existing conditions not only influence the clinical course during support but also impact short- and long-term survival rates (10). Understanding the effect of these variables is fundamental to optimizing ECMO candidate selection and developing more effective management strategies.

Despite growing international evidence regarding factors associated with outcomes in patients undergoing ECMO, a significant knowledge gap persists concerning the specific influence of comorbidities, particularly in middle-income settings such as Latin America. Less than 1% of cases in the Extracorporeal Life Support Organization (ELSO) international registry originate from South America, and estimates suggest that in countries like Brazil, only 10% of procedures are officially reported, reflecting substantial underreporting (11). Factors such as epidemiological characteristics, limited access to specialized resources, and variability in clinical practice can substantially modify the relationship between comorbidities and clinical outcomes. Given this context, an analysis tailored to our regional setting is warranted. Therefore, the objective of this study is to evaluate the association between comorbidities and 30-day mortality in patients undergoing ECMO, stratified by VA and VV modalities.

## Methods

### Study design

A cross-sectional cohort study was conducted on patients requiring extracorporeal circulation treated at a tertiary care center in Colombia between January 2019 and December 2024. The institution belongs to the ELSO international network and serves as a referral center for ECMO support in Bogotá, Colombia. The study adhered to the STROBE guidelines for observational studies (12).

### Eligibility criteria

We included patients aged ≥18 years requiring ECMO for respiratory or cardiac failure, in accordance with established international clinical guidelines. For VV ECMO, indications included severe hypoxemic respiratory failure (PaO_2_/FiO_2_ < 80 mmHg for >6 h or < 50 mmHg for >3 h despite protective mechanical ventilation), refractory respiratory acidosis (pH < 7.20), and a Murray score >3, consistent with the EOLIA trial criteria. For VA ECMO, patients presenting cardiogenic shock in stages D or E, as defined by the Society for Cardiovascular Angiography and Interventions (SCAI) classification, were included. Patients with missing survival data, incomplete variables, or unavailable medical records were excluded from the analysis.

### Variables and data collection

Data collection included sociodemographic variables (age, sex, weight, height), duration of mechanical ventilation prior to ECMO initiation, type of ECMO support, intensive care unit (ICU) length of stay, survival to discharge, and comorbidities assessed using the Charlson Comorbidity Index (CCI). Additionally, clinical severity scores, specifically APACHE II, were recorded within the first 24 h of ICU admission.

Data were extracted and verified directly from electronic health records (EHR) by at least two trained investigators to minimize transcription bias. Attention was given to ensuring the consistency of severity score calculations.

### Data analysis

Data were collected using the REDCap^®^ platform and subsequently exported to Microsoft Excel for analysis (13). Categorical variables were described as absolute frequencies and percentages. Continuous variables were expressed as mean ± standard deviation (SD) for normally distributed data, or as median and interquartile range (IQR) for non-normally distributed data. Normality was assessed using the Shapiro-Wilk test.

A “comorbidome” was generated to visualize associated comorbidities. In this graphical representation, the size of each bubble is proportional to disease prevalence, while proximity to the center reflects the strength of the association. This strength was numerically quantified as the inverse of the prevalence odds ratio (OR) for 28-day mortality (1/OR) (14). Graphic visualizations were performed using Python 3.12.

### Ethical considerations

The study was conducted in strict adherence to the ethical principles established in the Declaration of Helsinki, Resolution 8,430 of 1,993 of the Colombian Ministry of Health, and other applicable national and international guidelines for health research (15). Given its retrospective observational design, the study was classified as “no risk” as it involved no intervention or intentional modification of the participants' biological, physiological, psychological, or social variables, in accordance with Article 11 of Resolution 8,430.

## Results

A total of 436 patients were included, with a mean age of 43.7 (SD: 13.0) years and an overall mortality rate of 30%. Non-survivors were significantly older than survivors (48.2, SD: 11.9 vs. 41.7, SD: 13.0 years; *p* < 0.001). The duration of ECMO support in the ICU was significantly shorter in non-survivors [11 days (IQR 4.6–18.5)] compared to survivors [30 days (IQR 18.2–49.4); *p* < 0.001] (Table 1). Systolic blood pressure across the cohort was 107.4 ± 24.4 mmHg (105, SD: 22.0 in non-survivors vs. 108.3, SD: 25.3 in survivors). Diastolic blood pressure was 61.3 (SD: 14.3) mmHg (59.1, SD: 13.3 vs. 62.2, SD: 14.6), and mean arterial pressure was 76.7 (SD: 16.3) mmHg (74.4, SD: 14.7 vs. 77.6, SD: 16.8) (Table 1).

**Table 1:** General characteristics of the study population.

Variable	Total population *n* = 436	Non-survivors *n* = 133	Survivors *n* = 303	*P*-value
Age in years, mean (SD)	43.7 (13.01)	48.2 (11.89)	41.7 (13.01)	< 0,001
Age >65 years, *n* (%)	20 (4.5)	13 (10.2)	7 (2.3)	< 0,001
Male sex, *n* (%)	262 (60)	83 (66.4)	179 (59)	0,268
BMI, mean (SD)	28.1 (5.44)	28.3 (5.28)	28 (5.51)	1,000
Cause of ECMO *n* (%)				
Acute respiratory distress syndrome	257 (58.9)	75 (56)	182 (61)	0.745
Cardiogenic shock	163 (37.3)	50 (37)	113 (37)	
Others	16 (3.8)	8 (7)	8 (2)	
Venoarterial ECMO, *n* (%)	157 (36)	51 (38.4)	106 (34)	0,346
Venovenous ECMO, *n* (%)	279 (64)	82 (61.6)	197 (66)	0,815
Days of ECMO in ICU, me (IQR)	22.6 (12,45–39,16)	11 (4.56–18.5)	30 (18.22–49.36)	< 0.001
Days of mechanical ventilation, me (IQR)	15.1 (8,39–23,65)	11.2 (5.66–17.78)	16.3 (10–26.63)	< 0.001
Days of ECMO, me (IQR)	9.5 (4.35–18.65)	6.4 (1.7–12.24)	11.5 (4.9–22.44)	< 0,001
APACHE score, me (IQR)	10 (7–14)	11 (8–16)	10 (6–13.5)	0,006
Charlson index, me (IQR)	0 (0–1)	0 (0–1)	0 (0–1)	0,074
Total institutional stay days, me (IQR)	17,5 (7–28,75)	13 (5–19)	46 (33–60)	< 0.001

SD, Standard deviation; Me, Median; IQR, interquartile range; ICU, Intensive Care Unit; ECMO, Extracorporeal Membrane Oxygenation; BMI, Body Mass Index; APACHE, acute physiology and chronic health evaluation.

The median duration of mechanical ventilation was 15.1 days (IQR 8.4–23.7), with 11.2 days (IQR 5.7–17.8) for non-survivors and 16.3 days (IQR 10.0–26.6) for survivors. The median APACHE II score was 10 (IQR 7–14) overall [11 (IQR 8–16) vs. 10 (IQR 6–13.5)], and the Charlson Comorbidity Index was 0 (IQR 0–1) in both groups. Regarding ECMO modality, VA accounted for 36% of cases, while VV accounted for 64% (Table 1).

**Figure 1:**
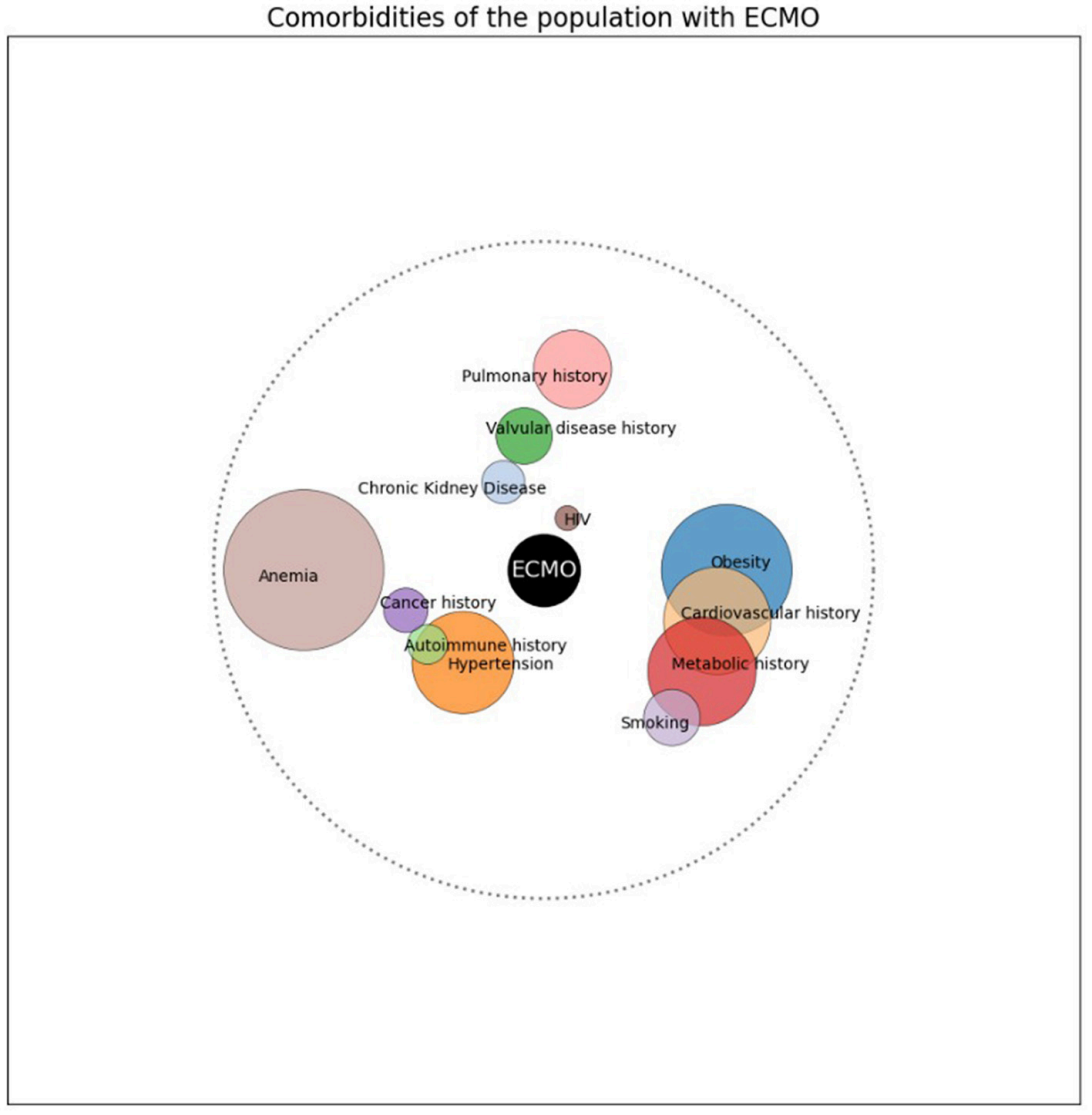
Comorbidome with extracorporeal membrane oxygenation.

In the overall ECMO cohort (*n* = 436), the most frequent comorbidities were anemia (47.7%), obesity (32.1%), and metabolic disorders (22.0%). Among non-survivors (*n* = 133), the highest prevalences were observed for anemia (44.3%), obesity (33.0%), and hypertension (24.8%), whereas survivors (*n* = 303) presented with anemia (49.1%), obesity (31.6%), and metabolic disorders (21.7%). The conditions most strongly associated with mortality were human immunodeficiency virus (HIV) infection (OR 3.60), chronic kidney disease (OR 2.12), and hypertension (OR 1.67) (Table 2) (Figure 1).

**Table 2:** Comorbidities with extracorporeal membrane oxygenation.

Variable	Total population *n* = 436	Non-survivors *n* = 133	Survivors *n* = 303	OR
Obesity	140 (32.1)	44 (33)	96 (31.6)	1.12
Alcohol	8 (1.8)	–	8 (2.6)	–
CKD	15 (3.4)	7 (5.2)	8 (2.6)	2.12
Hypertension	85 (19.4)	33 (24.8)	52 (17.1)	1.67
Cardiovascular history	94 (21.5)	30 (22.5)	64 (21.1)	1.14
Valvular disease history	26 (5.9)	10 (7.5)	16 (5.2)	1.51
Autoimmune history	13 (2.9)	5 (3.7)	8 (2.6)	1.49
Metabolic history	96 (22)	30 (22.5)	66 (21.7)	1.09
Pulmonary history	50 (4, 11)	15 (11.2)	35 (11.5)	1.01
Cancer history	16 (3, 6)	6 (4.5)	10 (3.3)	1.43
Smoking	26 (5.9)	8 (6)	18 (59.)	1.05
HIV	5 (1.1)	3 (2.2)	2 (0.6)	3.60
Anemia	208 (47.7)	59 (44.3)	149 (49.1)	0.86

OR: odds ratio; CKD: chronic kidney disease; HIV: human immunodeficiency virus.

**Figure 2:**
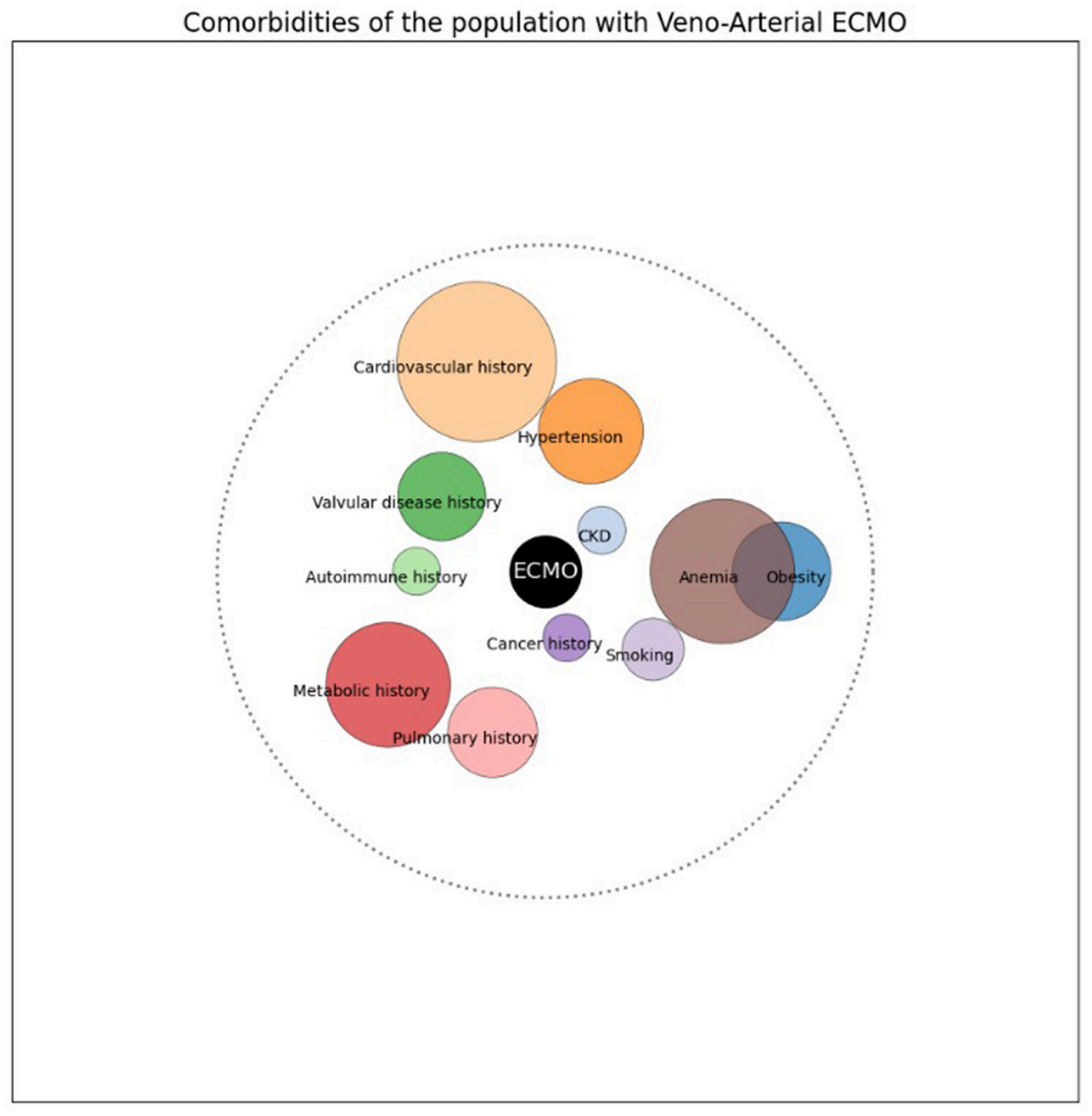
Comorbidome with veno-arterial extracorporeal membrane oxygenation.

In the VA ECMO group (*n* = 157), the leading comorbidities were cardiovascular history (50.3%), anemia (41.4%), and metabolic disorders (30.6%). Prevalence patterns were similar between non-survivors (*n* = 51)—who presented with cardiovascular history (49.0%), anemia (43.1%), and metabolic disorders (31.4%)—and survivors (*n* = 106), who showed cardiovascular history (50.9%), anemia (40.5%), and metabolic disorders (30.2%). The strongest predictors of mortality were chronic kidney disease (OR 2.92), history of cancer (OR 2.92), and valvular heart disease (OR 1.60) (Table 3) (Figure 2).

**Table 3:** Comorbidities with veno-arterial extracorporeal membrane oxygenation.

Variable	Total population *n* = 157	Non-survivors *n* = 51	Survivors *n* = 106	OR
Obesity	30 (19.1)	9 (17.6)	21 (19.8)	0.86
Alcohol	3 (1.9)	–	3 (2.8)	–
CKD	7 (4.5)	4 (7.8)	3 (2.8)	2.92
Hypertension	34 (21.7)	13 (25.5)	21 (19.8)	1.38
Cardiovascular history	79 (50.3)	25 (49)	54 (50.9)	0.92
Valvular disease history	24 (15.3)	10 (19.6)	14 (13.2)	1.60
Autoimmune history	7 (4.5)	3 (5.9)	4 (3.8)	1.59
Metabolic history	48 (30.6)	16 (31.4)	32 (30.2)	1.05
Pulmonary history	25 (15.9)	9 (17.6)	16 (15.1)	1.20
Cancer history	7 (4.5)	4 (7.8)	3 (2.8)	2.92
Smoking	12 (7.6)	5 (9.8)	7 (6.6)	1.53
HIV	1 (0.6)	1 (2)	–	–
Anemia	65 (41.4)	22 (43.1)	43 (40.5)	1.15

OR: Odds Ratio; CKD: Chronic Kidney Disease; HIV: Human Immunodeficiency Virus.

**Figure 3:**
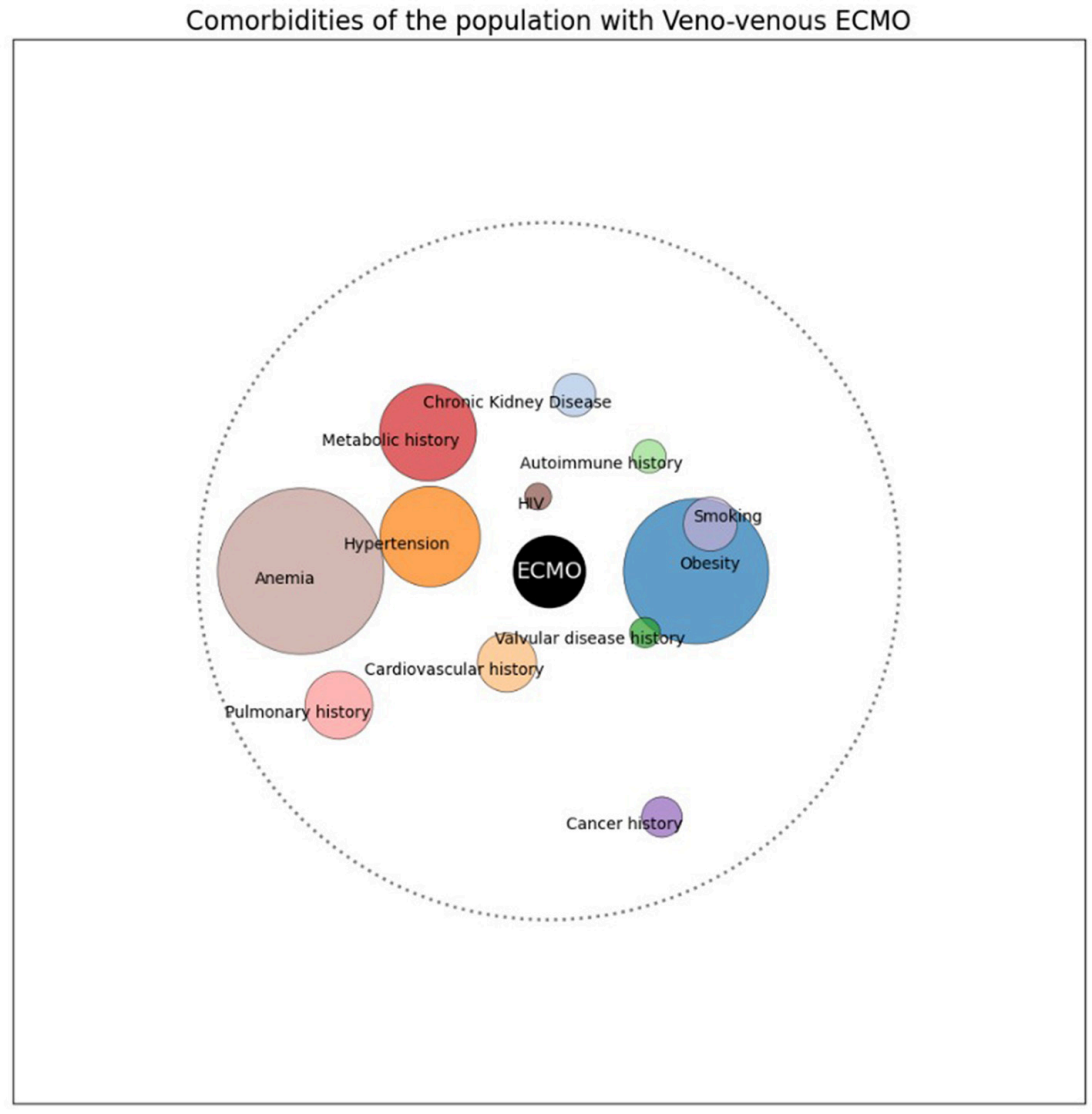
Comorbidome with veno-venous extracorporeal membrane oxygenation.

In the VV ECMO population, the most common comorbidities were anemia (54.1%), obesity (41.2%), and hypertension (19.7%). In non-survivors (*n* = 82), the most prevalent conditions were anemia (50.0%), obesity (45.1%), and hypertension (24.4%), while survivors (*n* = 197) presented with anemia (55.8%), obesity (39.6%), and metabolic disorders (18.3%). Mortality was most strongly associated with HIV infection (OR 2.44), cardiovascular history (OR 1.83), and valvular heart disease (OR 1.62) (Table 4) (Figure 3).

**Table 4:** Comorbidities with veno-venous extracorporeal membrane oxygenation.

Variable	Total population *n* = 279	Non-survivors *n* = 82	Survivors *n* = 197	OR
Obesity	115 (41.2)	37 (45.1)	78 (39.6)	1.25
Alcohol	5 (1.8)	–	5 (2.5)	–
CKD	10 (3.6)	3 (3.7)	7 (3.6)	1.03
Hypertension	55 (19.7)	20 (24.4)	35 (17.8)	1.49
Cardiovascular history	19 (6.8)	8 (9.8)	11 (5.6)	1.82
Valvular disease history	5 (1.8)	2 (2.4)	3 (1.5)	1.61
Autoimmune history	6 (2.2)	2 (2.4)	4 (2)	1.20
Metabolic history	51 (18.3)	15 (18.3)	36 (18.3)	1.00
Pulmonary history	25 (9)	6 (7.3)	19 (9.6)	0.74
Cancer history	9 (3.2)	2 (2.4)	7 (3.6)	0.67
Smoking	16 (5.7)	5 (6.1)	11 (5.6)	1.09
HIV	4 (1.4)	2 (2.4)	2 (1)	2.43
Anemia	151 (54.1)	41 (50.0)	110 (55.8)	0.74

OR: Odds Ratio; CKD: Chronic Kidney Disease; HIV: Human Immunodeficiency Virus.

## Discussion

This study evaluated the influence of pre-existing comorbidities on 30-day mortality in adult patients undergoing ECMO at a tertiary care center in Latin America, analyzing key differences between VA and VV modalities. The identification of comorbid conditions such as chronic kidney disease, hypertension, and HIV infection as being associated with higher mortality suggests that these factors should be carefully weighed during the evaluation of ECMO candidacy. Rather than serving as absolute contraindications, these variables may help guide clinical judgment, optimize resource allocation, and improve prognostic discussions with patients and families (16).

Our cohort presented an overall mortality rate of 30%, which lies at the lower end of the globally reported range (48.7%−76.38%) (17, 18) and below the meta-analyzed mortality of 37.7% for ARDS in VV ECMO (19). This comparatively favorable outcome may be explained by differences in patient selection, with our cohort likely representing individuals with a lower baseline comorbidity burden, as well as by local clinical practices and case-mix. Institutional selection criteria for ECMO candidacy may have favored patients with a higher likelihood of recovery, which should be considered when comparing these results with national or registry-based data.

The relatively low Charlson Comorbidity Index observed in our cohort likely reflects a selected population with a lower baseline burden of chronic disease (20). This finding may be explained by institutional selection criteria for ECMO candidacy, where patients with fewer comorbid conditions are preferentially considered to optimize outcomes in a resource-intensive therapy. Consequently, the comorbidity profile of our population may differ from that reported in broader or registry-based cohorts, potentially contributing to the lower observed mortality (21). This selection effect should be considered when interpreting both the comorbidome distribution and its association with clinical outcomes, as it may limit the extrapolation of our findings to more heterogeneous populations.

The most frequent comorbidities in the overall population were anemia, obesity, and metabolic disorders. The high prevalence of anemia is particularly relevant, as pre-existing anemia has been shown to be an independent predictor of worse outcomes and an increased risk of transfusion requirements (22), a factor that can exacerbate multiorgan failure in ECMO patients.

Notably, while obesity was one of the most prevalent comorbidities, especially in the VV modality (41.2%), it did not emerge as a primary predictor of overall mortality. This finding supports growing evidence regarding the ‘Obesity Paradox' in the Intensive Care Unit. Recent studies indicate that an elevated body mass index (BMI)—historically considered a relative contraindication—is not necessarily associated with increased mortality in patients undergoing ECMO (23) Furthermore, reviews such as that by Coccola et al. (24) suggest there is currently no evidence to support using BMI as an exclusion criterion for ECMO candidacy.

The most robust finding in our study is the strong association between HIV infection and overall mortality, with specific relevance in the VV-ECMO modality. This risk contrasts with emerging literature advocating against the exclusion of HIV patients. A systematic review by Rajsic et al (25) regarding patients newly diagnosed with HIV and ARDS showed that ECMO can serve as a successful bridge to lung recovery in up to 93% of cases. The discrepancy between our high mortality OR and the favorable outcomes reported by Rajsic et al. may be explained by our patient profile, which likely featured higher viral loads, severe immunodeficiency, or the development of sepsis—risk factors identified by Rajsic et al. Therefore, the decision to initiate support should be based on the patient's current immunological status and the presence of other comorbidities.

Regarding HIV, its association with ECMO mortality should be interpreted cautiously. Current evidence suggests that HIV infection alone does not directly determine outcomes; rather, prognosis is largely driven by the degree of immunosuppression, the presence of opportunistic infections, and complications such as sepsis (26, 27). In this context, HIV in our cohort likely reflects an underlying state of clinical vulnerability rather than acting as an independent causal factor. This interpretation aligns with prior studies in critically ill and immunocompromised populations, where outcomes are more closely related to associated conditions than to the diagnosis itself (28). Accordingly, within the comorbidome framework, HIV should be understood as part of a broader comorbidity profile rather than an isolated predictor.

On the other hand, chronic kidney disease (CKD) was the mortality predictor with the highest OR in the VA modality and a key factor overall. Pre-existing CKD serves as a marker of vascular and systemic frailty. In the context of VA-ECMO, hemodynamic instability and shock-associated hypoperfusion are the primary drivers of Acute Kidney Injury (AKI). In fact, studies such as that by Pérez-Garzón et al (29) have demonstrated that VA-ECMO therapy itself is significantly associated with the presence of Renal Angina, a risk marker for kidney injury progression. Pre-existing CKD, combined with the high risk of AKI associated with hemodynamic instability, creates a lethal synergy reflected in our high odds ratio.

Our results underscore the need for modality-specific risk stratification, demonstrated by the predictive value of valvular heart disease in both VA and VV cohorts. In VA-ECMO, baseline valvulopathy suggests advanced structural heart disease which, in the context of cardiogenic shock, hinders myocardial recovery despite ECLS offloading—a finding consistent with the high mortality reported in post-cardiotomy ECMO series (30).

In contrast, within the VV-ECMO cohort, where respiratory failure drives the indication, valvular disease and cardiovascular history (OR 1.83) serve as markers of reduced cardiopulmonary reserve. This impairment compromises the patient's ability to tolerate right ventricular afterload increases caused by ARDS-induced pulmonary hypertension, ultimately elevating the risk of adverse clinical outcomes.

### Strengths and limitations

Our findings should be interpreted considering several limitations. The retrospective, single-center design may limit generalizability and introduce potential selection bias. A distinctive strength of this work is the application of the Comorbidome concept within a cohort study in a middle-income country, addressing a specific knowledge gap in the ECMO literature. The relatively low Charlson Comorbidity Index values and the lower-than-expected mortality rate (29.8%) may reflect a cohort with a lower burden of comorbidities at baseline, possibly due to patient selection. Additionally, the analysis was intentionally designed as exploratory and descriptive, focusing on the characterization and visualization of comorbidity patterns through the comorbidome approach rather than on predictive or causal modeling. For this reason, no multivariable analysis (e.g., logistic regression including variables such as age, severity scores, or duration of mechanical ventilation) was performed. While this approach is consistent with the study objectives, it limits the ability to adjust for potential confounders and precludes establishing independent associations between variables and outcomes.

## Conclusion and clinical implications

In conclusion, this study confirms that comorbidities are critical determinants of prognosis in ECMO patients, and that their impact varies significantly according to the support modality. The presence of HIV infection, CKD, and hypertension are independently associated with increased mortality. We propose incorporating a comprehensive, modality-specific risk assessment into patient selection protocols, with particular attention to immunological status and renal function, to optimize clinical management. Further prospective, multicenter research in the Latin American region is required to validate these findings.

## Data Availability

The raw data supporting the conclusions of this article will be made available by the authors, without undue reservation.
